# Study on the effect of internet plus continuous nursing on functional recovery and medication compliance of patients with knee joint replacement

**DOI:** 10.1186/s13018-023-03907-1

**Published:** 2023-06-11

**Authors:** Yan Li, Zongyun Gu, Rende Ning, Hao Yin

**Affiliations:** Department of Joint Surgery, Hefei First People’s Hospital, Hefei, Anhui China

**Keywords:** "Internet + " continuity of care, Knee arthroplasty, Functional recovery, Medication compliance

## Abstract

**Objective:**

To evaluate the effect of "Internet + " continuity of care on postoperative functional recovery and medication compliance in patients with knee arthroplasty.

**Methods:**

In this retrospective study, 100 patients who underwent knee replacement in our hospital between January 2021 and December 2022 were recruited and assigned to receive routine care (routine group) or "Internet + " continuity of care (continuity group), with 50 patients in each group. Outcome measures included knee function, sleep quality, emotional state, medication compliance, and self-care ability.

**Results:**

Patients in the continuity group showed better knee function after discharge and during follow-up versus those in the routine group (P < 0.05). Continuity care resulted in significantly lower Pittsburgh Sleep Quality Index (PSQI), Self-Rating Anxiety Scale (SAS), and Self-Rating Depression Scale (SDS) scores versus routine care (P < 0.05). Patients in the continuity group showed higher treatment compliance, ability of daily living (ADL) scores, and nursing satisfaction than those in the routine group (P < 0.05).

**Conclusion:**

The "Internet + " continuity of care is highly feasible and can effectively promote the postoperative functional recovery of knee replacement patients, improve patients' medication compliance, sleep quality, and self-care ability, mitigate negative emotions, and provide enhanced home care.

## Introduction

The knee is the most complex structure in the human body and a joint with high demands on motor function. Knee injuries will result in severely compromised self-care ability, motor functions, and quality of life of patients [1]. Total knee arthroplasty (TKA) [2] provides pain relief, improves knee function and quality of life, and is currently the main clinical management for knee disorders [3]. However, treatment modalities based on knee replacement alone may be insufficient to fulfill the needs of all patients. A growing body of the literature suggests that the majority of patients after knee replacement have distinct psychological profiles characterized by catastrophic thoughts, dysfunctional disease perception, poor mental health, anxiety, and depression [4]. These factors may hinder physical recovery and lead to poor postoperative outcomes. Previous studies suggest that rehabilitation outcomes after knee arthroplasty are closely associated with nursing interventions and patient self-care [5, 6]. Therefore, individualized treatment based on the biopsychosocial health model is indicated for patients to improve the continuity of postoperative care and the effectiveness of rehabilitation exercises. In recent years, with the rapid development of the mobile internet, the application of mobile care in healthcare allows effective real-time information exchange and knowledge transfer, which overcomes the limitations of physical space and space [7], such as online platforms that allow patients to receive health education remotely [8, 9]. The present study was conducted to evaluate the effect of "Internet + " continuity of care on postoperative functional recovery and medication compliance in patients with knee arthroplasty.

## Materials and methods

### Participants

In this retrospective study, 100 patients who underwent knee replacement in our hospital between January 2021 and December 2022 were recruited and assigned to receive routine care (routine group) or "Internet + " continuity of care (continuity group), with 50 patients in each group. The study was approved by the ethics committee of our hospital, and all patients provided undersigned informed consent.

### Inclusion and exclusion criteria

#### Inclusion criteria

(1) all patients were aged over 18 years with normal cognitive ability and understanding; (2) all patients, regardless of gender, were scheduled for knee arthroplasty; (3) patients or family members in the two groups were able to use the Internet proficiently; (4) who had not participated in similar studies.

#### Exclusion criteria

(1) with heart, liver, or kidney or other serious uncontrollable diseases; (2) with intolerance to surgery or contraindications to relevant treatment; (3) with postoperative infection or serious complications; (4) with old or pathological fractures; (5) with other malignancies.

### Treatment methods

#### Total knee arthroplasty

Preoperative preparation was routinely performed to exclude contraindications to surgery and to improve relevant preoperative examinations. With the patient in the supine position, combined spinal and epidural anesthesia or general anesthesia was performed, followed by routine sterilization and draping. A 10–15 cm longitudinal incision was made in the anterior mid-knee to expose the joint by incising the medial support band and joint capsule along the medial aspect of the patella. The anterior–posterior and bicondylar lines were marked in preparation for osteotomy, the patella was turned laterally, and the hyperplasia, ligaments, and meniscus were removed. The force lines of the lower extremity and the balance of the extended and flexed knee gaps were assessed, and trial molds of the prosthesis were fitted. A suitable prosthesis was selected and fixed with bone cement, followed by hemostasis, irrigation of the wound, suturing layer-by-layer, and placement of a drainage tube [10, 11].

#### Routine nursing

The patients were regularly provided with health education to improve their disease awareness. The patients were assisted in various examinations, treatments, and rehabilitation exercises and were explained in detail about postoperative rehabilitation exercises. They were educated on the correct use of medications and provided with nutritional advice, instructed to observe and perform rehabilitation exercises before discharge, and were provided with regular hospital checkups and regular visits to inquire about rehabilitation conditions [12].

#### "Internet + " continuity of care

(1) Patient files were created, including general patient information, disease assessment, postoperative pathology results, key points of care and follow-up plans. (2) A public WeChat account and TKA patient WeChat group were created to share postoperative care measures and knowledge. (3) A continuity of care team network platform was established to train nursing staff on post-knee replacement rehabilitation nursing knowledge and network platform skills through an online learning platform. (4) Internet continuity of care programs was performed. After discharge, the patients were regularly provided with pain relief methods, complication prevention and response methods (skin, body temperature, circulation), use of antithrombotic drugs), prosthetic care methods (artificial knee joint materials, duration of use, daily maintenance, prosthetic prolapse treatment methods), rehabilitation training methods (walking, swimming, bicycling, etc.). Video assessment was conducted to timely observe and evaluate the recovery of patients, including knee joint activities, rehabilitation exercise methods, compliance with medical advice, pain, self-care, quality of life, mental and psychological disorders, and psychological status. Rehabilitation-related daily questions were addressed by the responsible medical staff or physicians, and general questions were summarized and answered collectively. The patients were instructed to perform gentle knee flexion and extension, sit-to-stand transition, hip extension, walking, walking up and down stairs and other active exercises, 10–15 min/time, 2–4 times/day. Muscle loading and weight-bearing walks were started in the 3rd month after discharge. Patients were encouraged to do housework and perform light exercises such as cycling and swimming. The psychological status of patients was regularly assessed, and appropriate psychological counseling was provided. The patients were reminded to visit the hospital regularly for review to understand their recovery. 5) The patients and their families were encouraged to communicate with each other to alleviate negative emotions, improve the self-service ability of patients and their families, help create a positive and active health mindset, and increase the compliance and initiative of patients and their families.

### Outcome measures

#### Knee function score

Knee function was assessed using the Hospital for Special Surgery Knee-Rating Scale (HSS), which includes pain (30 points), function (22 points), mobility (18 points), muscle strength (10 points), flexion deformity (10 points), and stability (10 points). The scores were rated at different time points after discharge (at discharge, one week after discharge, two weeks after discharge, one month after discharge, two months after discharge, and six months after discharge), and the scores were proportional to the patient's knee function, i.e., the higher the score the better the knee function.

#### Sleep quality and emotion

The Pittsburgh Sleep Quality Index (PSQI) was used to assess the patient's sleep quality, and the Self-Rating Anxiety Scale (SAS) and Self-Rating Depression Scale (SDS) were used to assess the patient's anxiety and depression. PSQI scores were inversely proportional to sleep status, i.e., higher scores indicated poorer sleep quality, and SAS and SDS scores were positively proportional to psychological status, i.e., higher scores indicated higher levels of anxiety and depression.

#### Medication compliance

The medication compliance questionnaire (Cronbach's alpha coefficient 0.847, retest validity 0.858), which was self-administered by our hospital, was used for assessment. The total score was 100, with above 80 for complete compliance, 60–80 for basic compliance, and below 60 for non-compliance. Total compliance rate = (number of complete compliance cases + number of basic compliance cases)/total number of cases.

#### Ability of daily living

The ability of daily living (ADL) was used to assess the patient's self-care ability. The total score was 100, and the score was proportional to the patient's self-care ability, i.e., the higher the score, the better the ability of daily living.

#### Patient satisfaction

Home-made Nursing Satisfaction Questionnaires (including attitude of nursing staff, efficiency of nursing staff, and explanation of diseases by nursing staff) (divided into highly satisfied, satisfied, less satisfied, and unsatisfied) were used to understand the satisfaction of patients in both groups. Total satisfaction = (number of highly satisfied cases + number of satisfied cases)/total number of cases.

### Statistical analysis

GraphPad Prism 8 was used to process the images, and SPSS 26.0 software was used to organize and statistically analyze the data. The measurement data were expressed as mean ± standard deviation (mean ± s) and analyzed using the t test. The count data were expressed as rate(%) and processed using the chi-square test. P < 0.05 was used as a cut-off for statistical significance.

## Results

### Patient characteristics

There were 50 patients in the routine group, including 12 males, 38 females, aged 50–83 (64.16 ± 10.02) years, and 50 patients in the continuity group, including 13 males, 37 females, aged 50–81 (66.30 ± 7.74) years. The patient characteristics between the two groups were comparable (P > 0.05). (Table 1).

**Table 1:** Patient characteristics

	Routine (n = 50)	Continuity (n = 50)	*t*	*P*
*Sex*				
Male	12 (24.00)	13 (26.00)	–	–
Female	38 (76.00)	37 (74.00)	–	–
*Age (years)*				
–	50–83	50–81	–	–
Mean	64.16 ± 10.02	66.30 ± 7.74	1.195	0.235
*BMI (kg/m*^*2*^*)*				
–	19–26	19–26	–	–
Mean	22.84 ± 0.59	22.94 ± 0.68	1.111	0.268
*Reason for surgery*				
Knee Osteoarthritis	24 (48.00)	25 (50.00)	–	–
Rheumatoid arthritis	26 (52.00)	25 (50.00)	–	–
*Surgical site*				
Left knee	25 (50.00)	28 (56.00)	–	–
Right knee	25 (50.00)	22 (44.00)	–	–
*Duration of knee lesion (yrs)*				
–	1–9	1–9	–	–
Mean	4.88 ± 1.21	5.02 ± 1.03	0.881	0.379
*Time-lapses after discharge (d)*				
–	1–45	1–45	–	–
Mean	35.34 ± 1.65	35.08 ± 1.77	1.074	0.284
*Duration of education (years)*				
–	6–21	6–21	–	–
Mean	12.27 ± 3.31	12.01 ± 3.45	0.544	0.587

BMI: body mass index

### Knee function scores

**Fig. 1:**
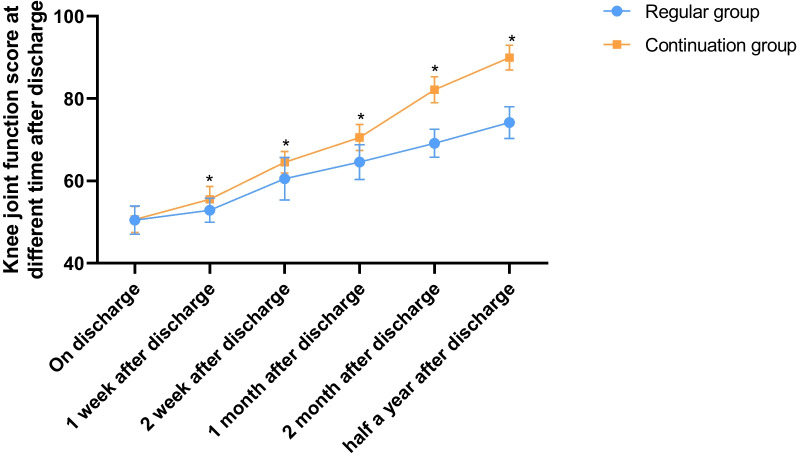
Knee function scores. *Note*: * indicates statistical differences (P < 0.05) when compared with the routine group

Patients in the continuity group had knee function scores at discharge (50.48 ± 3.45), one week after discharge (52.88 ± 2.89), two weeks after discharge (60.51 ± 5.15), one month after discharge (64.56 ± 4.22), two months after discharge (69.14 ± 3.41), and six months after discharge (74.16 ± 3.84). Patients in the continuity group had knee function scores at discharge (50.65 ± 3.17), one week after discharge (55.54 ± 3.15), two weeks after discharge (64.54 ± 2.63), one month after discharge (70.54 ± 3.15), two months after discharge (82.14 ± 3.15), and six months after discharge (89.94 ± 3.01). Patients in the continuity group showed better knee function after discharge and during follow-up versus those in the routine group (P < 0.05). (Fig. 1).

### Quality of care and emotions

There was no statistically significant difference between the two groups in the sleep quality and emotional status before the intervention (P > 0. 05). After the intervention, PSQI, SAS, and SDS scores in the continuity group (7.01 ± 0.66, 34.16 ± 3.15, 40.14 ± 2.44) were significantly lower than those in the routine group (9.98 ± 0.75, 48.45 ± 3.14, 50.15 ± 3.14) (P < 0.05). (Table 2).

**Table 2:** PSQI, SAS, and SDS scores

	Routine (n = 50)	Continuity (n = 50)	*t*	*P*
*Before intervention*				
PSQI	13.54 ± 0.88	13.49 ± 0.91	0.395	0.693
SAS	57.45 ± 2.56	57.89 ± 2.14	1.319	0.189
SDS	59.45 ± 3.14	59.21 ± 3.42	0.517	0.606
*After intervention*				
PSQI	9.98 ± 0.75*	7.01 ± 0.66*	29.728	< 0.001
SAS	48.45 ± 3.14*	34.16 ± 3.15*	32.129	< 0.001
SDS	50.15 ± 3.14*	40.14 ± 2.44*	25.172	< 0.001

*Indicates statistical differences between the pre- and post-treatment data; PSQI: Pittsburgh Sleep Quality Index; SAS: Self-Rating Anxiety Scale; SDS: Self-Rating Depression Scale

### Medication compliance

In the routine group, there were 16 cases of complete compliance, 23 cases of basic compliance and 11 cases of non-compliance, while in the continuity group, there were 21 cases of complete compliance, 27 cases of basic compliance and 2 cases of non-compliance. Patients in the continuity group showed higher treatment compliance than those in the routine group (P < 0.05) (Table 3).

**Table 3:** Medication compliance

	Routine (n = 50)	Continuity (n = 50)	*x*^2^	*P*
Complete compliance	16 (32.00)	21 (42.00)	–	–
Basic compliance	23 (45.00)	27 (54.00)	–	–
Non-compliance	11 (22.00)	2 (4.00)	–	–
Total compliance rate	39 (78.00)	48 (96.00)	7.162	< 0.001

### Self-care ability

No statistically significant differences were found in the sleep quality and emotional status of the two groups before the intervention (P > 0.05). After the intervention, the ADL score of the continuity group (83.45 ± 7.22) was significantly higher than that of the routine group (75.15 ± 8.15) (P < 0.05) (Table 4).

**Table 4:** Self-care ability

	Routine (n = 50)	Continuity (n = 50)	*t*	*P*
Before intervention	61.15 ± 10.51	61.35 ± 9.94	0.138	0.890
After intervention	75.15 ± 8.15	83.45 ± 7.22	7.623	< 0.001

*Indicates statistical differences between the pre- and post-treatment data

### Patient satisfaction

**Fig. 2:**
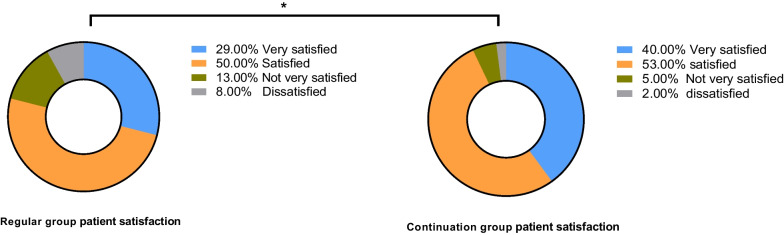
Patient satisfaction. *Note*: * indicates statistical differences between the pre- and post-treatment data

In the routine group, 15 patients were highly satisfied, 25 were satisfied, 6 were less satisfied, and 4 were dissatisfied. In the continuity group, 20 patients were highly satisfied, 27 were satisfied, 2 were less satisfied, and 1 were dissatisfied. Total patient satisfaction was significantly higher in the continuity group (94.00%) than in the routine group (80.00%) (P < 0.05) (Fig. 2).

## Discussion

TKA provides joint repair and reconstruction and improves joint function for patients with knee joint disease, and its clinical effectiveness has been well-established by the medical community. However, due to the lack of knowledge about knee replacement rehabilitation, most patients are prone to anxiety and panic after discharge, and the interruption of professional rehabilitation care after discharge results in difficulties in carrying out standardized and effective rehabilitation education, causing poor motor performance and compromised recovery [13]. Therefore, scientific and effective rehabilitation care contributes to the recovery of knee function and improves the mobility of the knee joint after surgery.

The results of the present study showed that patients in the continuity group showed better knee function after discharge and during follow-up versus those in the routine group. Continuity care resulted in significantly lower PSQI, SAS, and SDS scores versus routine care. Patients in the continuity group showed higher treatment compliance, ADL scores, and nursing satisfaction than those in the routine group. These results suggested that "Internet + " continuity care provides bidirectional communication between nursing and patients, promotes patients' recovery, and improves the quality of life. Continued care services were provided to patients after discharge through WeChat groups or WeChat public accounts, including methods of pain relief after knee replacement, methods of complication prevention and response, methods of prosthesis maintenance, and methods of rehabilitation training to improve patients' compliance with rehabilitation education. Also, patients were instructed to perform rehabilitation exercises, such as knee flexion, muscle strength, flexibility, and weight-bearing capacity, to facilitate the recovery of knee function. Patients regularly reported feedback to medical staff on the effects of rehabilitation exercises and obtained professional and personalized advice and suggestions to improve patient compliance and satisfaction [14]. Patients were also provided with comprehensive nursing advice such as nutritional advice and rehabilitation exercises to provide strong support for post-discharge care [15, 16]. Communication and encouragement enhance the patient's initiative to promote self-rehabilitation management and recovery of joint function, thereby improving their ADL.

Continuous care is an extension of inpatient care, providing uninterrupted medical services for discharged patients, and enables nursing staff to understand patient recovery and issues with patient behavior and care processes after discharge. However, in previous related studies, telephone follow-up was conducted for patients with total knee arthroplasty, and the results showed that it could improve patients' rehabilitation after discharge, but there was little improvement in postoperative rehabilitation exercises, joint rehabilitation activities, and living conditions. Mere reliance on telephone follow-up is clearly insufficient for exhaustive and intuitive follow-up and is of limited use for home rehabilitation. Web-based continuity of care, however, allows caregivers to communicate with patients and provide advice on home rehabilitation, reduces psychological, physical, and social barriers, enhances rehabilitation outcomes, and also prevents complications [17, 18]. In this study, Internet-based continuity of care was applied to the care of patients with TKA, and the intervention was extended from the hospital to the home through video assessments, demonstrations, training, and question-and-answer sessions to provide comprehensive nursing interventions for patients. The results showed that "Internet + " continuity of care could effectively improve knee function, reduce patients' negative emotions, and significantly improve patients' home rehabilitation, which is consistent with the results of prior studies [19, 20].

Notwithstanding the strength of "Internet + " continuity of care, several feasible protocols concerning functional rehabilitation have been reported before. Stefani L et al. [21] pointed out that individually prescribed home-based exercise programs were cost effective and safe and resulted in modest improvements in body composition, strength, and total body water distribution with little to no adverse effect on cardiac function. Paravlic AH et al. [22] reported that motor imagery (MI) practice plus physical therapy improves both objective and subjective measures of patients' physical function after TKA and facilitates transfer of MI strength task on functional mobility. In addition, supervised training and home exercises improved long-term outcome in patients with ankylosing spondylitis versus educational-behavioral program or no intervention [23]. Masiero S et al. revealed that following knee surgery, rehabilitation can dramatically affect the postoperative course and the final outcomes of the procedure. And the authors systematically reviewed the current literature comparing clinical outcomes of home-based and outpatient supervised rehabilitation protocols following knee surgery and concluded that supervised and home-based protocols did not show an overall significant difference in the outcomes achieved within the studies reviewed. Rompe JD et al. [24] indicated that both corticosteroid injection and home training were significantly less successful than shock wave therapy at 4-month follow-up. Corticosteroid injection was significantly less successful than home training or shock wave therapy at 15-month follow-up. Speculatively, the above-mentioned programs might result in favorable outcomes combined with "Internet + " continuity of care in the current study.

## Conclusion

The "Internet + " continuity of care is highly feasible and can effectively promote the postoperative functional recovery of knee replacement patients, improve patients' medication compliance, sleep quality and self-care ability, mitigate negative emotions, and provide enhanced home care.

Our study has the strength of a systematic and comprehensive assessment of "Internet + " continuity of care in postoperative functional recovery of knee replacement patients; however, important limitations should be outlined. The sample studied comprised a select and relatively small number of participants, facts which affect the generalizability of the findings. Due to the design characteristics, causality was not pursued. Hence, a prospective and controlled trial in a larger sample is needed to strengthen our hypothesis. In addition, due to multiple factors, we didn’t reveal comparison of major post-OP medications like analgesics and psychologic drugs as anti-anxiety ones between both groups, which would possibly bias our results toward the null. Therefore, ongoing studies with comparison of major post-OP medications are warranted.

## Prospects

In recent years, with the development and progress of science and technology, the Internet is the inevitable trend of the future. The "Internet + " continuity of care breaks through the time and space limitations of traditional extended care, with the advantages of economy, practicality and rapidity, and facilitates patient recovery, which demonstrates great potential for clinical promotion.

## Data Availability

The datasets used and analyzed during the current study are available from the corresponding author on reasonable request.
